# Comparative Evaluation of Advanced Irrigant Activation Protocols on Hydrogel Removal From Standardized Artificial Lateral Canals: An In Vitro Study

**DOI:** 10.1002/cre2.70442

**Published:** 2026-08-26

**Authors:** Alfonso Acerra, Dina Abdellatif, Francesco Giordano, Davide Mancino, Gianrico Spagnuolo, Mariangela Cernera, Alfredo Iandolo

**Affiliations:** ^1^ Department of Medicine, Surgery and Dentistry University of Salerno Salerno Italy; ^2^ CHU Besançon Besançon France; ^3^ Laboratoire SINERGIES EA 4662 University of Marie & Louis Pasteur Besançon France; ^4^ Department of Endodontics and Restorative Dentistry University of Marie & Louis Pasteur Besançon France; ^5^ Department of Endodontics, Faculty of Dental Medicine Strasbourg University Strasbourg France; ^6^ Pôle de Médecine et Chirurgie Bucco‐Dentaire, Hôpitaux Universitaires de Strasbourg Strasbourg France; ^7^ INSERM UMR_S 1121, Biomaterials and Bioengineering Strasbourg France; ^8^ Department of Neurosciences, Reproductive and Odontostomatological Sciences University of Naples “Federico II” Napoli Italy; ^9^ Therapeutic Dentistry Department, Institute for Dentistry Sechenov University Moscow Russia

**Keywords:** Dual Rinse HEDP, internal heating, passive ultrasonic irrigation (PUI), sodium hypochlorite (NaOCl), sonic activation

## Abstract

**Objective:**

To compare irrigant activation protocols for hydrogel removal from standardized artificial lateral canals.

**Materials and Methods:**

One hundred and seventy‐six 3D‐printed resin blocks reproducing a standardized root canal with two lateral canals were used. The lateral canals were filled with gelatin–hyaluronan hydrogel as an organic‐debris surrogate. Blocks were randomized to 16 groups (A1–A8, B1–B8; *n* = 11/group). Group A protocols used 3% sodium hypochlorite (NaOCl), whereas Group B protocols used 3% NaOCl with Dual Rinse HEDP. The primary outcome was net specimen mass loss (W2−W3), determined gravimetrically. Optical microscopy provided descriptive support. Non‐parametric analyses were applied to the gravimetric outcome (*α* = 0.05).

**Results:**

Saline controls and conventional syringe‐irrigation groups showed no detectable net mass loss at the balance readability of 0.1 mg. Net mass loss increased from sonic and ultrasonic activation to protocols combining internal heating with activation. Groups A7 and B7 produced the greatest median net mass loss (0.60 mg). Descriptive microscopy indicated greater hydrogel removal from the smaller apical lateral canal in Groups A6–A8 and B6–B8 than in Groups A3–A5 and B3–B5, with the greatest removal in A7 and B7. No significant gravimetric differences were detected between corresponding Group A and Group B protocols (*p* > 0.05).

**Conclusions:**

Within the limitations of this thermosensitive hydrogel model, protocols combining internal heating with manual dynamic, ultrasonic or sonic activation were associated with greater net mass loss and microscopy‐based hydrogel removal than conventional irrigation or single‐mode activation. Although consistent with the temperature‐dependent increase in NaOCl tissue‐dissolving activity, chemical dissolution could not be distinguished from thermally induced hydrogel softening and mechanical displacement. No protocol completely removed hydrogel from the smaller apical lateral canal, and the findings should not be extrapolated directly to clinical root canal systems.

## 1 Introduction

Effective disinfection of the root canal system remains a primary objective of endodontic therapy (Gomes et al. 2023). Despite advances in mechanical instrumentation, complete removal of organic debris and microbial biofilm from complex anatomical structures, including lateral canals, apical ramifications, and isthmuses, remains a significant clinical challenge (Zehnder 2006; Iandolo et al. 2012). These anatomical intricacies often harbor persistent microorganisms, contributing to treatment failure and post‐treatment disease (Siqueira et al. 2018). Conventional syringe irrigation, although widely used, has been shown to provide limited irrigant penetration and fluid exchange in anatomically complex regions, particularly in the apical third and lateral extensions (Pantaleo et al. 2022; Teja et al. 2023). As a result, various irrigant activation strategies have been introduced to enhance the chemical and mechanical actions of irrigants, including sonic activation, passive ultrasonic irrigation (PUI), internal heating, and laser‐assisted irrigation (Virdee et al. 2018). These methods aim to improve fluid dynamics, shear stress, cavitation, and chemical reactivity, thereby promoting deeper irrigant penetration and more effective debridement (Yilmaz et al. 2020). Among commonly used irrigants, sodium hypochlorite (NaOCl) remains the gold standard due to its potent antimicrobial activity and unique tissue‐dissolving properties (Zou et al. 2010). However, its effectiveness is influenced by several factors, including concentration, temperature, exposure time, and method of activation.

Temperature is an important determinant of the tissue‐dissolving activity of sodium hypochlorite. Experimental studies using pulp tissue have shown that heated sodium hypochlorite produces faster tissue dissolution than the same solution used at lower temperatures and that intracanal heating may further enhance this effect. Therefore, the improved removal of organic material observed with heated irrigation protocols may reflect increased chemical reactivity and tissue‐dissolving capacity, in addition to changes in fluid dynamics (Abdellatif, Plotino et al. 2025). Recently, Dual Rinse HEDP, which permits continuous chelation during NaOCl irrigation, has been proposed to facilitate smear‐layer management while retaining the organic‐tissue‐dissolving action of NaOCl. In a simulated endodontic environment, Ballal et al. (2021) reported that HEDP did not impair the tissue‐dissolving or gelatinolytic effect of ultrasonically activated NaOCl.

The use of stained hydrogels in transparent artificial canal systems is an established experimental approach. Macedo et al. (2014) described a reproducible methodology for assessing hydrogel removal from lateral morphological features of the root canal. Subsequent studies used related transparent‐resin or three‐dimensionally printed models to investigate the effects of irrigant streaming, cavitation, activation method, tip design, insertion depth and canal geometry on material removal from lateral canals and isthmuses (Robinson et al. 2018; Swimberghe et al. 2019; Donnermeyer et al. 2023, 2024; Bürklein et al. 2024). Aydın et al. employed a particularly close experimental model comprising three‐dimensionally printed resin blocks with two lateral canals and a gravimetric outcome (Aydın et al. 2025). Retsas et al. (2022), using artificial lateral canals containing a dual‐species microbial biofilm, similarly demonstrated that activation improved material removal but did not achieve complete biofilm eradication. Although some previous studies described their material as a “biofilm‐mimicking hydrogel,” the acellular gelatin–hyaluronan hydrogel used in the present investigation was regarded solely as an organic‐debris surrogate.

Against this established methodological background, the incremental contribution of the present study lies in the systematic comparison of two irrigant regimens—3% NaOCl and 3% NaOCl supplemented with Dual Rinse HEDP—across a common matrix of conventional syringe irrigation, internal heating, manual dynamic activation, sonic activation, and ultrasonic activation. Particular attention was given to protocols combining internal heating with subsequent activation and to material removal from a small apically positioned lateral canal. Therefore, the present study aimed to compare the hydrogel‐removal performance of these irrigation protocols in standardized artificial lateral canals, with particular emphasis on the small‐diameter lateral canal located 1 mm from the apical foramen.

### 1.1 Null Hypothesis (H_0_)

The null hypothesis tested was that no significant differences would be observed among the various irrigation and activation protocols, nor between the two irrigant regimens (3% NaOCl alone and 3% NaOCl supplemented with Dual Rinse HEDP), in terms of hydrogel removal from artificial lateral canals.

## 2 Materials and Methods

### 2.1 Sample Size Calculation

An a priori sample‐size estimation was performed using G*Power software (version 3.1.9.4; Heinrich Heine University Düsseldorf, Düsseldorf, Germany). The primary outcome was net specimen mass loss (W2−W3, mg), measured using an analytical balance. As G*Power does not provide a direct procedure for estimating sample size for the Kruskal–Wallis test with 16 independent groups, a one‐way fixed‐effects omnibus ANOVA was used as the initial planning approximation.

Related studies had reported measures of dispersion for hydrogel‐removal outcomes in artificial canal systems, including Aydın et al. (2025) who used three‐dimensionally printed resin blocks containing two lateral canals and a gravimetric outcome). Other studies reported dispersion for optical hydrogel‐removal outcomes in related artificial‐canal configurations (Robinson et al. 2018; Swimberghe et al. 2019; Donnermeyer et al. 2023; Bürklein et al. 2024). However, these estimates were not used to derive the planning effect size for the present 16‐group comparison because the studies differed in canal geometry, irrigant concentration, activation protocols, outcome definition, and group structure. A standardized effect size of *f* = 0.40 was therefore adopted as a pragmatic planning assumption rather than as an empirically derived estimate. With *α* = 0.05, 90% power and 16 independent groups, the parametric calculation indicated a minimum total sample size of 161 specimens. Under the normal‐theory planning assumption, the asymptotic relative efficiency of the Kruskal–Wallis test relative to one‐way ANOVA was taken as 0.955. The ANOVA‐derived minimum was therefore divided by 0.955 (161/0.955 = 168.6) and rounded up to 169 specimens. A total of 176 resin blocks was included, corresponding to 11 specimens per group and permitting balanced allocation across the 16 experimental groups. This calculation should be regarded as a pragmatic planning approximation rather than as an exact prospective power calculation for the Kruskal–Wallis analysis.

Each resin block was treated as a single independent experimental unit and assigned to only one protocol to avoid carry‐over effects and preserve standardized anatomy (main canal with two lateral canals). This approach enabled controlled comparisons between two irrigant regimens (3% NaOCl alone vs. 3% NaOCl supplemented with Dual Rinse HEDP) and among activation techniques, while minimizing confounding arising from anatomical variability.

### 2.2 Artificial Canal Design and Fabrication

The artificial canal system was digitally designed using computer‐aided design (CAD) software (AutoCAD, version 2024; Autodesk Inc., San Rafael, CA, USA) and fabricated using a professional‐grade three‐dimensional (3D) printer (Form 3B; Formlabs, Somerville, MA, USA) with a printing resolution of 0.05 mm. A biocompatible, optically transparent resin (BioMed Clear Resin; Formlabs) was selected to allow direct visualization of irrigant penetration and hydrogel removal during experimental procedures.

**Figure 1:**
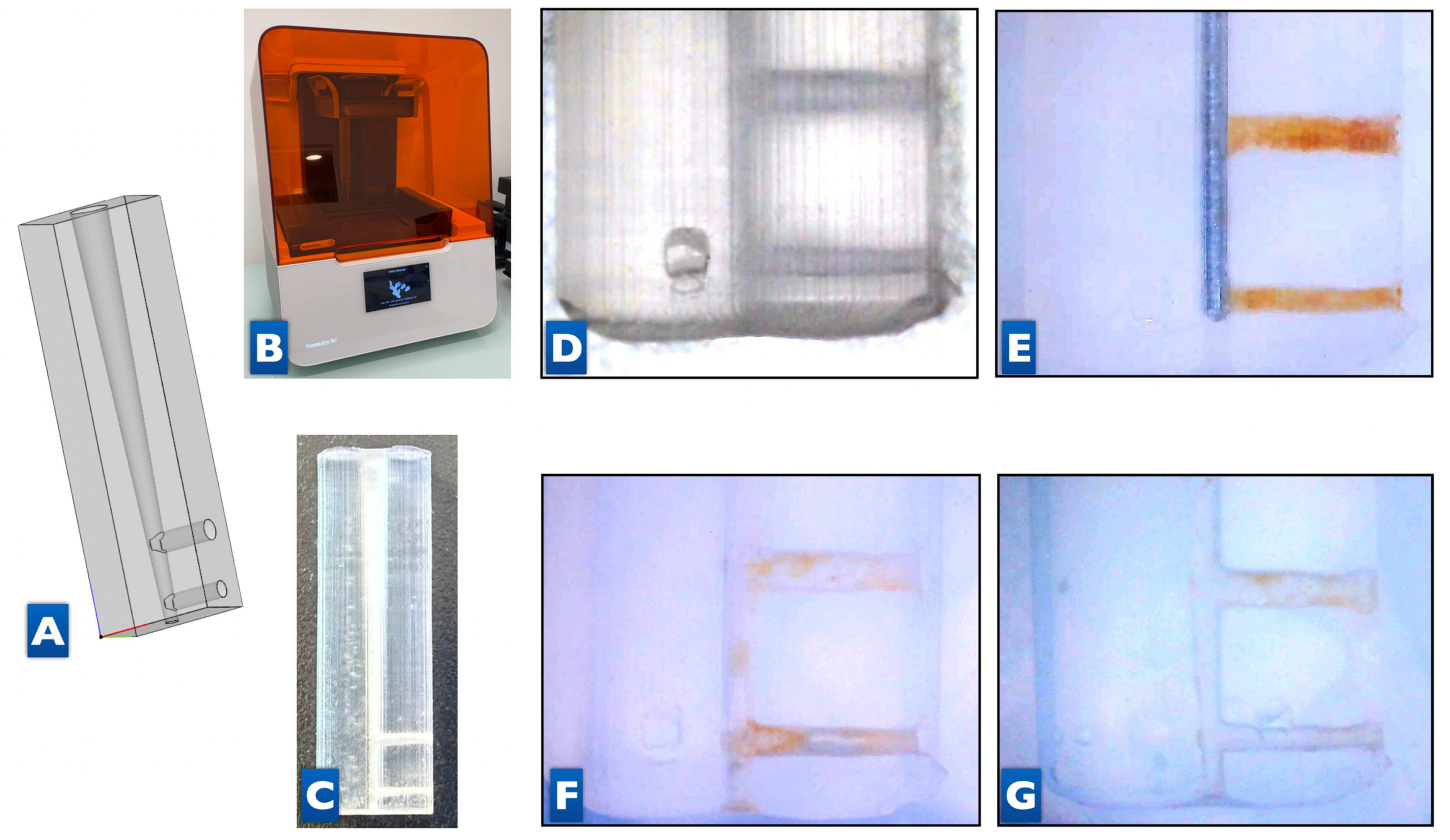
(A) Artificial canal system digitally designed using computer‐aided design (CAD) software. (B) 3D printer used for model fabrication. (C) Artificial canal system printed in resin. (D) Two cylindrical lateral canals empty. (E) Two cylindrical lateral canals filled with hydrogel. (F, G) Representative images showing hydrogel removal after the irrigant activation techniques.

**Figure 2:**
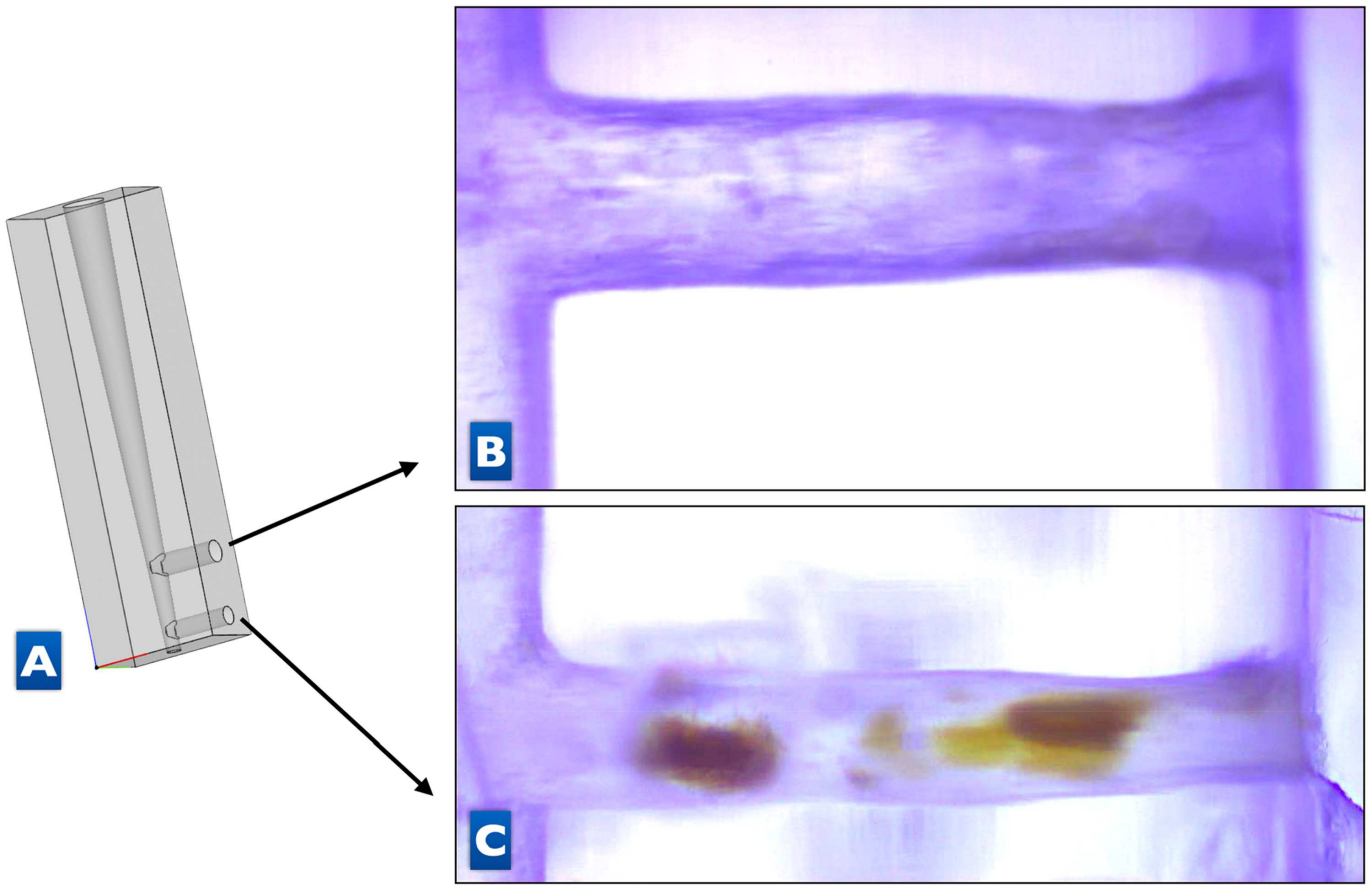
(A) Artificial canal system digitally designed using computer‐aided design (CAD) software. (B) Representative image of the larger lateral canal without hydrogel, showing complete hydrogel removal after irrigant activation. (C) Representative image of the smaller lateral canal with residual hydrogel remaining after the irrigant activation phase.

The main canal was designed with an apical diameter of 0.30 mm, a 6% taper, and a total working length of 16 mm, simulating the geometric characteristics of a prepared human root canal. Two cylindrical lateral canals were incorporated into the model to replicate clinically relevant anatomical complexities. The first lateral canal measured 2.2 mm in length and 0.25 mm in diameter, and was positioned 1 mm from the apical terminus. In contrast, the second lateral canal measured 2.2 mm in length and 0.60 mm in diameter, and was located 3 mm from the apical terminus (Figures 1 and 2).

This standardized artificial canal configuration enabled controlled, reproducible, and anatomically consistent experimental conditions for the comparative evaluation of irrigant activation protocols.

#### 2.2.1 Preparation and Application of the Hydrogel Organic‐Debris Surrogate

The hydrogel used as a standardized organic‐debris surrogate within the artificial lateral canals was prepared by dissolving 3 g of gelatin (Merck, New Jersey, USA) and 0.06 g of hyaluronan (95% sodium hyaluronate; Fisher Scientific, Waltham, MA, USA) in 45 mL of distilled water under continuous stirring at 50°C. To facilitate visual inspection and ensure accurate placement within the lateral canals, 0.25 g of red food dye (KTC, Wednesbury, UK) was added to the solution, yielding a uniformly colored hydrogel (Aydın et al. 2025).

Preliminary handling tests showed that the gelatin–hyaluronan hydrogel underwent a temperature‐dependent transition from a workable liquid state to a solid gel within approximately 1 min at room temperature (21°C). It was therefore maintained at 30°C before placement to retain sufficient fluidity for standardized filling of the artificial lateral canals. These preliminary observations were intended solely to establish suitable handling and placement conditions and did not constitute a rheological characterization or determination of the hydrogel melting temperature.

The liquid hydrogel was carefully introduced into both artificial lateral canals using hand files under magnification with an operating microscope. Visual inspection was used to confirm complete and homogeneous filling and to exclude specimens with visible air voids or incomplete hydrogel placement. After placement, the hydrogel was allowed to remain within the lateral canals for at least 1 min at room temperature (21°C) to cool and solidify before randomization and application of the irrigation protocols.

Following hydrogel application, D.A., who was not involved in the irrigation procedures, generated a computer‐based allocation sequence using Randomizer.org (https://www.randomizer.org/). The 176 resin blocks were subsequently allocated to the 16 experimental groups (A1–A8 and B1–B8), with 11 specimens per group. Because the assigned irrigation and activation procedures were visibly distinguishable, group allocation could not be concealed from M.C., the intervention operator, during delivery of the experimental protocols.

### 2.3 Irrigation and Activation Protocols

All irrigation procedures were performed by M.C., a single experienced operator. Blinding of the intervention operator was not feasible because the irrigants, activation devices and procedures were visibly distinguishable among the experimental groups. To minimize performance variability, irrigant volume, delivery rate, activation duration, tip position, number of cycles, and environmental conditions were standardized across the applicable protocols. To better simulate clinical operating conditions, the apex and lateral canals of each specimen were sealed with modeling wax, which was maintained throughout the irrigation procedures and removed only immediately before the final weighing.

In Groups A2–A8, 3% sodium hypochlorite (NaOCl) was used alone. In Groups B2–B8, 3% NaOCl supplemented with Dual Rinse HEDP was used; the mixture was freshly prepared immediately before use in accordance with the manufacturer's instructions. Groups A1 and B1 received sterile saline only. Control groups (A1 and B1) were irrigated exclusively with sterile saline solution.

For all protocols, irrigant delivery was performed using a 30‐gauge side‐vented closed‐ended needle (CanalPro Sideport Tips; Coltene/Whaledent Inc., Cuyahoga Falls, OH, USA), positioned 1 mm short of the working length, and connected to a syringe pump (Pilote A2; Fresenius Vial SAS, Brézins, France) to ensure a constant flow rate of 2 mL/min. The total irrigant volume was standardized to 6 mL per specimen.

*Group A1 and B1 – Control (Saline)*

Sterile saline solution was delivered at 2 mL/min for a total volume of 6 mL. After irrigation, the canal was dried with sterile paper points and immediately weighed.

*Group A2 – Conventional Syringe Irrigation (NaOCl)*

*Group B2 – Conventional Syringe Irrigation (Dual Rinse HEDP)*

The irrigant solution was delivered at a constant flow rate of 2 mL/min, reaching a total volume of 6 mL. Subsequently, the canal was flushed with 2 mL of sterile saline, dried with sterile paper points, and weighed.

*Group A3 – Sonic Activation (EDDY) (NaOCl)*

*Group B3 – Sonic Activation (EDDY) (Dual Rinse HEDP)*

Sonic activation was performed using a non‐cutting polyamide tip (#25) (EDDY; Dentsply Sirona, Ballaigues, Switzerland) mounted on an air‐driven handpiece (KaVo Kerr, Detroit, MI, USA). The tip was positioned 2 mm short of the working length, and each 2 mL aliquot of irrigant was activated for 20 s at maximum intensity, resulting in a total activation time of 60 s. After irrigation, canals were flushed with 2 mL of sterile saline, dried with sterile paper points, and weighed.

*Group A4 – Passive Ultrasonic Irrigation (PUI) (NaOCl)*

*Group B4 – Passive Ultrasonic Irrigation (PUI) (Dual Rinse HEDP)*

Ultrasonic activation was achieved using a titanium ultrasonic file (#25.00) powered by an ultrasonic device (Ultra Smart AI; Coxo, Foshan, China). The file was positioned 2 mm short of the working length, and each 2 mL aliquot was activated for 20 s, yielding a total activation time of 60 s. After irrigation, the canal was flushed with 2 mL of sterile saline, dried, and weighed.

*Group A5 – Internal Heating (NaOCl)*

*Group B5 – Internal Heating (Dual Rinse HEDP)*

Thermal activation of the irrigant was performed using an X‐fine Buchanan heat plugger tip (30/.04) connected to an Elements Obturation Unit (Kerr Endodontics, Orange, CA, USA) and set at 180°C. The selected device setting represents the temperature of the heat source and not the temperature necessarily reached by the irrigant or hydrogel within the artificial lateral canals. Local temperature changes at the lateral‐canal level were not measured. The plugger was inserted 3 mm short of the working length, without contacting the canal walls, and activated for 8 s after every 1 mL of irrigant delivery, resulting in a total of 6 heating cycles for 6 mL of solution. After irrigation, the canal was flushed with 2 mL of sterile saline, dried, and weighed.

*Group A6 – Internal Heating* + *MDA Activation (NaOCl)*

*Group B6 – Internal Heating* + *MDA Activation (Dual Rinse HEDP)*

Each heating cycle (8 s) was immediately followed by 60 s of manual dynamic activation using a gutta‐percha cone (35/.06; Coltene/Whaledent Inc.). The cone was positioned 2 mm short of the working length and activated using controlled vertical push–pull movements within the canal. This activation sequence was repeated after each 1 mL aliquot, yielding a total of 6 combined activation cycles for a final irrigant volume of 6 mL. Following irrigation, the canals were flushed with 2 mL of sterile saline, dried with sterile paper points, and weighed.

*Group A7 – Internal Heating + Passive Ultrasonic Irrigation (NaOCl)*

*Group B7 – Internal Heating + Passive Ultrasonic Irrigation (Dual Rinse HEDP)*

Each 8‐s internal heating cycle was immediately followed by 20 s of ultrasonic activation. This combined protocol was applied after each 1 mL aliquot, for a total of 6 activation cycles and 6 mL of irrigant. After irrigation, the canal was flushed with 2 mL of sterile saline, dried, and weighed.

Group A8 – Internal Heating + Sonic Activation (EDDY) (NaOCl)

Group B8 – Internal Heating + Sonic Activation (EDDY) (Dual Rinse HEDP)

Each 8‐s internal heating cycle was immediately followed by 20 s of sonic activation. Sonic activation was performed using a non‐cutting polyamide tip (#25) (EDDY; Dentsply Sirona, Ballaigues, Switzerland) mounted on an air‐driven handpiece (KaVo Kerr, Detroit, MI, USA). The tip was positioned 2 mm short of the working length, and each 1 mL aliquot of irrigant was activated for 20 s at maximum intensity. This sequence was repeated after each 1 mL aliquot, yielding a total of six combined activation cycles for a final irrigant volume of 6 mL. After irrigation, the canals were flushed with 2 mL of sterile saline, dried with sterile paper points, and weighed.

### 2.4 Weighing Procedure

The gravimetric measurements were performed by A.A., who was not involved in generating the allocation sequence or delivering the irrigation procedures and was blinded to experimental‐group allocation during weighing. Each specimen was identified by a numerical code that did not disclose the irrigant or activation protocol.

All mass measurements were performed using an analytical balance (Pioneer PA224C; OHAUS Corporation, Parsippany, NJ, USA) with a readability of 0.0001 g (0.1 mg). At each weighing stage, three consecutive stable readings were obtained and averaged. Because the balance had a readability of 0.1 mg, the resulting mean was retained to the nearest 0.1 mg before calculation of W2–W3. Consequently, the gravimetric differences were expressed on a discrete 0.1 mg measurement grid, and smaller positive or negative mass fluctuations could not be resolved. The repeated readings were used to confirm measurement stability and were not treated as independent observations. W1 represented the mass of the empty, dry resin block before hydrogel placement; W2 represented the mass of the same block after hydrogel placement and solidification; and W3 represented its mass after completion of the irrigation protocol, removal of the modeling wax and paper‐point drying.

The paired difference W2–W3 was calculated arithmetically as the net specimen mass loss occurring during the experimental procedure. No threshold based on visual or microscopic evidence of material removal was applied. Because W2–W3 may include changes in hydrogel mass as well as changes in resin moisture content, residual fluid, trace wax residue, or other non‐hydrogel components, it was not regarded as a chemically specific measurement of hydrogel dissolution. A calculated value of 0.0 mg indicated no detectable difference between W2 and W3 at the 0.1 mg readability of the balance; it should not be interpreted as evidence of an exact zero mass change, because smaller positive or negative fluctuations could not be resolved.

As a geometric plausibility check, the combined nominal volume of the two cylindrical lateral canals was calculated from the design dimensions as *π*(0.125 mm)^2^(2.2 mm) + *π*(0.30 mm)^2^ (2.2 mm) = 0.730 mm^3^. The density of the prepared hydrogel was not measured; therefore, its exact nominal mass could not be determined. For contextual comparison only, assuming an aqueous‐hydrogel density of 1.00–1.05 mg/mm^3^ yields an estimated hydrogel mass of approximately 0.73–0.77 mg.

The W2 measurement was recorded before application of the modeling wax. The wax was applied only after W2 had been obtained and was carefully removed before W3 was recorded. The canal was dried using sterile paper points of the same size until the final paper point appeared visually dry. The same drying criterion and the same interval between drying and W3 measurement were applied to all specimens.

All weighing procedures were conducted on a dedicated vibration‐free laboratory bench under controlled environmental conditions to minimize the potential effects of mechanical disturbances, air currents, and temperature fluctuations.

### 2.5 Optical Microscopy Evaluation

Following the irrigation protocols, the lateral canals were examined using an optical microscope (Optika B‐290 TB, Optika S.r.l., Ponteranica, Bergamo, Italy) equipped with a high‐resolution digital camera.

Following image acquisition, the microscopy images were assigned numerical codes that did not disclose the irrigant or activation protocol and were presented in a random order. The coded images were quantitatively assessed by A.I., who was not involved in generating the allocation sequence or delivering the irrigation procedures and did not have access to the coding key during image analysis. A.I. was therefore an independent outcome assessor and was blinded to experimental‐group allocation during the microscopic assessment.

Both lateral canals were analyzed, with particular attention to the smaller apically located lateral canal. Digital images were acquired at standardized magnification and illumination settings to ensure reproducibility.

Quantitative image analysis was performed using ImageJ software (National Institutes of Health, Bethesda, MD, USA). All images were acquired using the same magnification, illumination, exposure, and background conditions. For each specimen, the lateral‐canal lumen was delineated as a predefined region of interest, and the area occupied by the stained hydrogel was measured before and after irrigation. Images were converted to 8‐bit greyscale and processed using the same predefined thresholding procedure to distinguish the stained hydrogel from the surrounding transparent resin. The thresholding criteria were applied independently of the experimental group and were not adjusted according to the expected outcome.

The percentage of hydrogel removal was calculated as the relative reduction in hydrogel‐covered area compared to the initial condition, using the following formula:

$\text{Hydrogel}\;\text{removal}\left(\%\right)=\left[\left(A_{0}-A_{1}\right)/A_{0}\right]\times 100$

where *A*_0_ represents the initial hydrogel‐filled area and *A*_1_ the residual hydrogel area after irrigation.

The percentage reduction was used to generate group‐level descriptive summaries. Because the specimen‐level percentage‐reduction dataset was not available in the archived material for re‐analysis, microscopy was treated as a supportive descriptive outcome and was not subjected to inferential statistical analysis. Before the definitive assessment, A.I. underwent a calibration session using representative images that were not included in the final analysis. The calibration images were assessed repeatedly to standardize the delineation of the region of interest and application of the thresholding procedure.

### 2.6 Statistical Analysis

The principal inferential analyses were performed using SPSS software (version 27.0; IBM Corp., Armonk, NY, USA). The effect‐size calculations and percentile bootstrap confidence intervals reported in the revised analyses were computed using Python 3.12.13 (Python Software Foundation), SciPy 1.17.0 and NumPy 2.3.5, with 2000 resamples and a fixed random‐number seed of 20260731.

The primary gravimetric outcome was net specimen mass loss (mg), calculated as W2–W3. W1 was recorded as the empty‐block baseline to document the weighing sequence; W1 alone was not analyzed as an outcome or used to adjust the primary W2–W3 analysis. Because W1 preceded the experimental procedures, it could not account for subsequent changes in non‐hydrogel mass resulting from irrigation, thermal cycling, fluid sorption, residual moisture, wax handling or drying. All measurements were expressed in milligrams (mg).

The initial hydrogel‐associated mass (W2–W1) was examined as a supporting baseline variable and compared among the 16 experimental groups using the Kruskal–Wallis test. Because a small between‐group difference in initial hydrogel‐associated mass was detected, an exploratory sensitivity analysis was also performed by normalizing net specimen mass loss to the initial hydrogel‐associated mass: (W2–W3)/(W2–W1). This analysis was undertaken solely to assess the robustness of the primary between‐group pattern and did not replace W2–W3 as the primary outcome, because normalization cannot exclude changes in non‐hydrogel specimen mass occurring between W2 and W3.

The distribution of the primary gravimetric outcome was assessed using the Shapiro–Wilk test and by visual inspection of histograms and Q–Q plots. The gravimetric data showed non‐normality and heteroscedasticity across groups; therefore, non‐parametric statistical methods were applied.

Descriptive statistics were reported as medians, first and third quartiles (Q1–Q3), and minimum and maximum values for each experimental group (A1–A8 and B1–B8).

Zero and tied values were retained as recorded without substitution or imputation, and tie corrections were applied in the non‐parametric analyses.

Inter‐group comparisons were conducted using the Kruskal–Wallis test to evaluate overall differences among the 16 experimental groups. When statistically significant differences were detected, pairwise comparisons were performed using the Mann–Whitney *U* test, with Bonferroni correction applied to adjust for multiple testing.

To investigate differences between the two main irrigation protocols (Group A vs. Group B), pooled comparisons were conducted using the Mann–Whitney *U* test. Additionally, subgroup analyses were conducted to compare the corresponding activation protocols between Group A and Group B (e.g., A1 vs. B1, A2 vs. B2, etc.).

The magnitude of the overall group effect was estimated using eta‐squared for the Kruskal–Wallis test, calculated as *η* ^2^ *H* = (*H* − *k* + 1)/(*N* − *k*), where *H* is the Kruskal–Wallis statistic, *k* is the number of groups, and *N* is the total sample size. Effect sizes for pairwise comparisons were calculated as *r* = *Z*/√*N*, where Z is the tie‐corrected standardized Mann–Whitney statistic and *N* is the number of observations included in the comparison. The sign of *r* indicates the direction of the difference, whereas absolute *r* values of approximately 0.10, 0.30, and 0.50 were interpreted as small, moderate, and large effects, respectively. Unadjusted percentile bootstrap 95% confidence intervals were calculated using 2000 resamples; these intervals were not adjusted for multiplicity. The complete pairwise gravimetric effect‐size estimates and their confidence intervals are reported in Table S1.

The microscopy findings were summarized descriptively. No distributional testing, between‐group inferential analysis, effect‐size calculation or bootstrap confidence interval was performed for the microscopy outcome because the specimen‐level measurements were not available in the archived dataset.

Graphical representation of the gravimetric data was performed using box‐and‐whisker plots showing medians, interquartile ranges, and dispersion for each experimental group.

All statistical tests were two‐tailed, and the level of significance was set at *α* = 0.05.

## 3 Results

The initial hydrogel‐associated mass (W2–W1) ranged from 0.8 to 0.9 mg, with a median of 0.8 mg in every experimental group. Nevertheless, the distribution of this baseline variable differed among the 16 groups (*H*(15) = 27.03, *p* = 0.028, *η* ^2^ *H* = 0.075, unadjusted percentile bootstrap 95% CI 0.024–0.304), indicating that initial hydrogel loading was not completely balanced. In an exploratory sensitivity analysis, normalizing net specimen mass loss to the initial hydrogel‐associated mass did not materially alter the overall findings. The normalized outcome remained significantly different among the 16 groups (*H*(15) = 161.00, *p* < 0.001, *η* ^2^ *H* = 0.912, unadjusted percentile bootstrap 95% CI 0.904–0.936), whereas the pooled comparison between Groups A and B remained non‐significant (*U* = 3858.5, *p* = 0.968, *r* = −0.003, unadjusted percentile bootstrap 95% CI −0.148 to 0.147).

**Figure 3:**
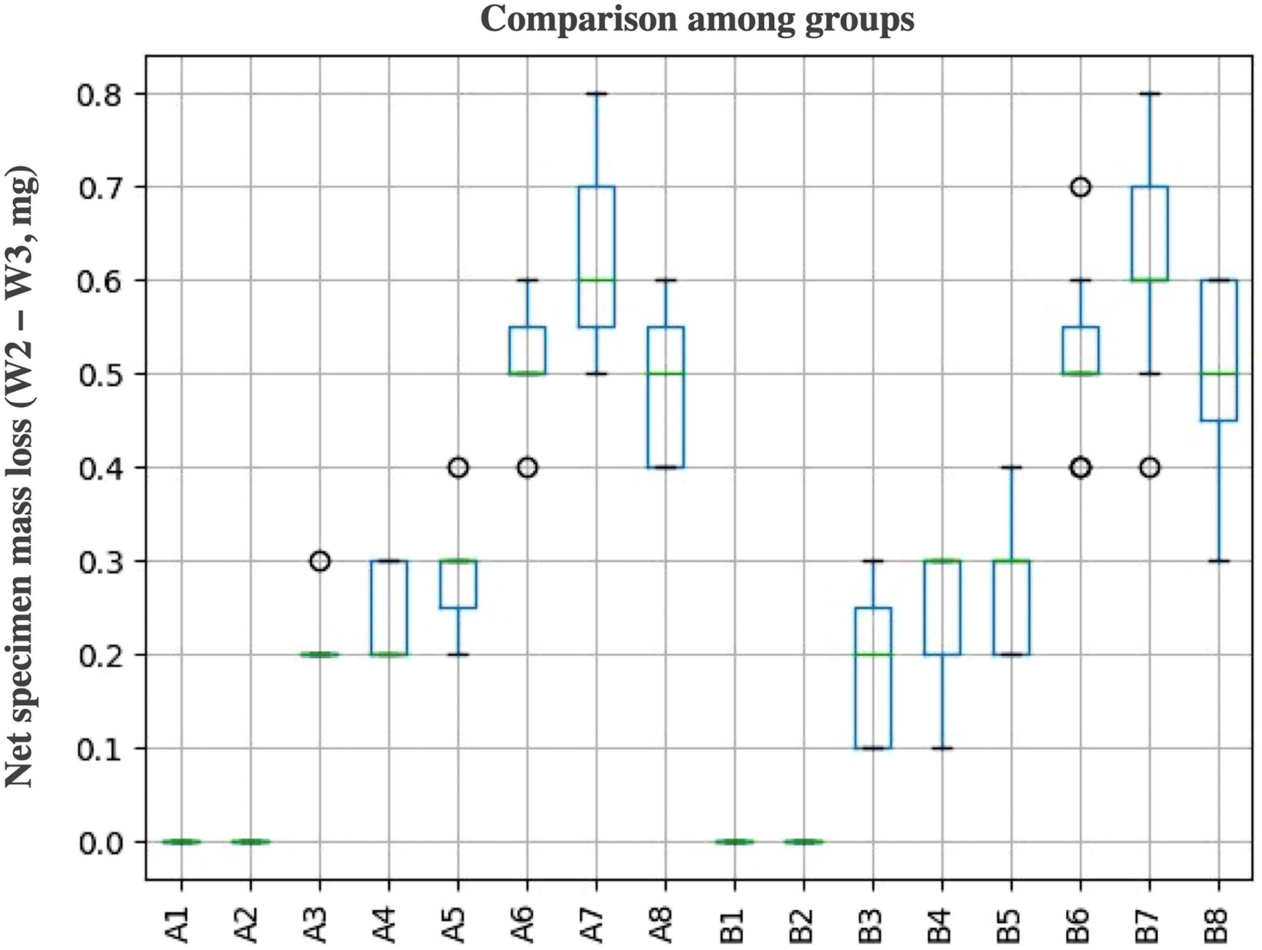
Box‐and‐whisker plots of net specimen mass loss (W2–W3, mg) across the 16 experimental groups. Boxes represent the interquartile range, center lines represent the median, whiskers represent values within 1.5 times the interquartile range, and individual symbols indicate outlying observations. Group A, 3% NaOCl; Group B, 3% NaOCl supplemented with Dual Rinse HEDP; 1, saline control; 2, conventional syringe irrigation; 3, sonic activation; 4, passive ultrasonic irrigation; 5, internal heating; 6, internal heating plus manual dynamic activation; 7, internal heating plus passive ultrasonic irrigation; 8, internal heating plus sonic activation.

Gravimetric data are reported as net specimen mass loss (mg), calculated as W2–W3. The distribution of the gravimetric outcome across the 16 experimental groups is illustrated in Figure 3, whereas descriptive statistics are summarized in Table 1. In the saline‐control and conventional syringe‐irrigation groups (A1, A2, B1, and B2), W2 and W3 were identical at the retained 0.1 mg measurement precision for all specimens, resulting in recorded net specimen mass‐loss values of 0.0 mg. These values indicate no detectable change at the readability of the balance rather than the exact absence of smaller mass fluctuations. The activated protocols produced greater net specimen mass loss, with the greatest median values observed in A7 and B7 (0.6 mg), followed by A6/B6 and A8/B8.

**Table 1:** Descriptive statistics for net specimen mass loss (W2–W3, mg) in the 16 experimental groups.

Group	Median (mg)	Q1–Q3 (mg)	Minimum (mg)	Maximum (mg)
A1	0.0	0.0–0.0	0.0	0.0
A2	0.0	0.0–0.0	0.0	0.0
A3	0.2	0.2–0.2	0.2	0.3
A4	0.2	0.2–0.3	0.2	0.3
A5	0.3	0.25–0.3	0.2	0.4
A6	0.5	0.5–0.55	0.4	0.6
A7	0.6	0.55–0.7	0.5	0.8
A8	0.5	0.4–0.55	0.4	0.6
B1	0.0	0.0–0.0	0.0	0.0
B2	0.0	0.0–0.0	0.0	0.0
B3	0.2	0.1–0.25	0.1	0.3
B4	0.3	0.2–0.3	0.1	0.3
B5	0.3	0.2–0.3	0.2	0.4
B6	0.5	0.5–0.55	0.4	0.7
B7	0.6	0.6–0.7	0.4	0.8
B8	0.5	0.45–0.6	0.3	0.6

*Note:* Data are presented as the median, first and third quartiles (Q1–Q3), and minimum and maximum values. The overall between‐group effect was large (Kruskal–Wallis *η* ^2^ *H* = 0.911, unadjusted percentile bootstrap 95% CI 0.903–0.934; 2000 resamples). Complete pairwise effect‐size estimates are reported in Table S1. The measurements represent net specimen mass loss and should not be interpreted as a chemically specific measure of hydrogel dissolution. Recorded values of 0.0 mg indicate that no difference between W2 and W3 was detectable at the 0.1 mg readability of the balance; they should not be interpreted as demonstrating the exact absence of smaller positive or negative mass fluctuations. Medians, minima, and maxima are reported to one decimal place, consistent with the 0.1 mg readability of the balance; interpolated quartiles are reported to two decimal places where necessary. The additional decimal place in interpolated quartiles does not indicate measurement precision beyond the 0.1 mg readability of the balance.

The maximum recorded net specimen mass loss was 0.8 mg in Groups A7 and B7. This value approached or slightly exceeded the estimated hydrogel mass of approximately 0.73–0.77 mg derived from the nominal lateral‐canal geometry and an assumed aqueous‐hydrogel density of 1.00–1.05 mg/mm^3^.

Re‐examination of the specimen‐level gravimetric data confirmed equal group totals for A4/B4 (2.7 mg), A5/B5 (3.1 mg), A6/B6 (5.7 mg), A7/B7 (6.8 mg), and A8/B8 (5.4 mg). However, the corresponding specimen‐level distributions were not identical. The equal totals and resulting means arose from different combinations of observations on the discrete 0.1 mg measurement grid rather than from duplicated values. Complete specimen‐level W2–W3 measurements are provided in Table S2.

The Kruskal–Wallis test revealed a significant difference in net specimen mass loss among the 16 experimental groups (*H*(15) = 160.74, *p* < 0.001), with a large overall effect (*η* ^2^ *H* = 0.911, unadjusted percentile bootstrap 95% CI 0.903–0.934). The saline‐control and conventional syringe‐irrigation groups showed significantly lower net specimen mass loss than the activated protocols (Bonferroni‐adjusted *p* ≤ 0.003), with consistently large pairwise effects (| *r* | = 0.910–0.957). Furthermore, A3 and B3 showed significantly lower net specimen mass loss than A6–A8 and B6–B8 (Bonferroni‐adjusted *p* ≤ 0.015), with large pairwise effects (| *r* | = 0.819–0.907). The pooled analysis did not detect a statistically significant difference between Groups A and B (*U* = 3876.5, *p* = 0.989, *r* = 0.001, unadjusted percentile bootstrap 95% CI −0.143 to 0.150), and none of the comparisons between corresponding Group A and Group B protocols was statistically significant after Bonferroni correction. Complete pairwise estimates, including non‐significant comparisons, are provided in Table S1.

### 3.1 Optical Microscopy Results

The microscopy findings were interpreted descriptively. The saline‐control and conventional syringe‐irrigation groups (A1, A2, B1, and B2) showed no visually detectable hydrogel removal from either artificial lateral canal. Groups A3–A5 and B3–B5 showed extensive removal from the larger coronal lateral canal but only limited removal from the smaller apical lateral canal. Greater removal from both canals was observed in Groups A6–A8 and B6–B8, with the most pronounced removal observed in A7 and B7. Because the specimen‐level percentage‐reduction dataset was unavailable for re‐analysis, no measures of dispersion or inferential statistics are reported for this supportive outcome.

## 4 Discussion

The null hypothesis for the primary gravimetric outcome was rejected because a significant overall difference was observed among the experimental protocols. However, not all pairwise comparisons remained significant after adjustment for multiple testing. Within the constraints of the present model, the combined heating and activation protocols generally produced the greatest descriptive hydrogel‐removal values.

The present study compared the hydrogel‐removal performance of multiple irrigation protocols in a standardized artificial root canal system containing two lateral canals of different dimensions and positions. Particular attention was paid to the smaller lateral canal, located 1 mm from the apical foramen, which is one of the most critical anatomical regions in endodontic therapy.

The gravimetric findings demonstrated a progressive increase in net specimen mass loss across the tested protocols. Conventional syringe irrigation and saline irrigation produced no detectable mass change under the conditions of the present model. Sonic, ultrasonic, and internally heated protocols produced greater net specimen mass loss. The descriptive microscopy findings similarly suggested greater material removal from the smaller apical lateral canal with the combined protocols (A6–A8 and B6–B8), particularly A7 and B7. Because the microscopy outcome was evaluated descriptively, no inferential conclusion can be drawn from these supportive observations.

The present findings extend an established body of research using stained hydrogels and transparent artificial canal models. Macedo et al. (2014) established the underlying hydrogel‐removal methodology, whereas Robinson et al. (2018) demonstrated that streaming, cavitation and instrument position influence removal from lateral morphological features. Swimberghe et al. (2019) subsequently compared sonic, ultrasonic and laser activation in an isthmus model, and Donnermeyer et al. (2023, 2024) and Bürklein et al. (2024) demonstrated that activation method, canal configuration, tip design and insertion depth affect hydrogel removal in three‐dimensionally printed systems. Aydın et al. (2025) provide the closest methodological comparison because they used a three‐dimensionally printed two‐lateral‐canal resin model and gravimetric assessment. Accordingly, neither the artificial model nor the general observation that activation can improve hydrogel removal should be considered novel. The present study instead extends this literature by comparing NaOCl with NaOCl supplemented with HEDP across a common matrix that included internal heating and combined activation protocols.

Although no statistically significant differences were detected between corresponding Group A and Group B protocols, these findings should not be interpreted as evidence of equivalence between 3% NaOCl alone and 3% NaOCl supplemented with Dual Rinse HEDP. The study was not designed as an equivalence investigation: no equivalence margin was pre‐specified, and the sample‐size calculation addressed an omnibus difference among the 16 experimental groups. Therefore, the results indicate only that no between‐regimen difference was detected under the present experimental conditions and sample size.

The absence of a detected difference between the two irrigant regimens may also be considered in relation to the findings of Ballal et al. (2021), who reported that HEDP did not impair the tissue‐dissolving or gelatinolytic effect of ultrasonically activated NaOCl in simulated endodontic environments. The present findings are compatible with preservation of NaOCl activity after HEDP addition under the tested conditions. Nevertheless, they do not establish equality or equivalence between the two irrigant regimens.

Although the present investigation assessed removal of an acellular hydrogel from artificial lateral canals rather than smear‐layer removal from instrumented dentine, related studies illustrate that root canal cleanliness depends on both irrigant delivery and solution chemistry. Rajamanickam et al. (2022) detected no significant difference in debris or smear‐layer removal between an automated irrigation device and conventional syringe irrigation, indicating that device automation alone does not necessarily improve canal‐wall cleanliness. In a systematic review, Teja et al. (2022) reported that the evaluated herbal agents were generally less effective than EDTA for smear‐layer removal, underscoring the importance of chelating chemistry and the limited evidence supporting alternative agents used alone. These outcomes are not directly equivalent to hydrogel removal from artificial lateral canals. In the present protocols, Dual Rinse HEDP should therefore be interpreted as a continuous‐chelation adjunct within a NaOCl‐based regimen rather than as a substitute for NaOCl‐mediated organic‐tissue dissolution or irrigant activation.

The paired weighing design reduced the influence of static differences in resin‐block mass by using each specimen as its own baseline. Based on the nominal lateral‐canal geometry, the combined hydrogel volume was approximately 0.730 mm^3^, corresponding to an estimated mass of approximately 0.73–0.77 mg when an aqueous‐hydrogel density of 1.00–1.05 mg/mm^3^ is assumed. The maximum net specimen mass loss of 0.8 mg was therefore approximately 0.03–0.07 mg higher than this contextual estimate. Because the hydrogel density and actual printed canal volumes were not measured independently, this comparison remains approximate. Nevertheless, a W2−W3 value at or above the estimated total hydrogel mass cannot be attributed confidently to hydrogel removal alone. Irrigation, thermal cycling, wax application and removal, paper‐point drying, resin moisture changes, residual intracanal fluid and heat‐related evaporation may have altered the non‐hydrogel mass of individual specimens. In the absence of hydrogel‐free sham specimens, the magnitude and direction of these contributions cannot be quantified. Accordingly, the gravimetric outcome should be interpreted as net specimen mass loss rather than as a chemically specific measurement of hydrogel dissolution.

A small but statistically significant between‐group difference was also detected in the initial hydrogel‐associated mass (W2–W1). Although all group medians were 0.8 mg and the overall range was limited to 0.8–0.9 mg, this finding indicates that the amount of material initially available for removal was not completely uniform across groups. However, an exploratory analysis normalizing W2–W3 to W2–W1 produced essentially the same overall between‐group pattern as the primary analysis. This sensitivity analysis supports the robustness of the main group comparison but does not eliminate the potential contribution of non‐hydrogel mass changes occurring between W2 and W3.

Furthermore, the 0.1 mg readability of the balance meant that smaller positive or negative mass fluctuations could not be resolved. The tied zero values and absence of observed dispersion in Groups A1, A2, B1, and B2 therefore reflect the readability of the balance and should not be interpreted as evidence that handling, wetting, and drying produced no physical mass variation. This measurement constraint reduced the sensitivity of the gravimetric assessment for very small mass changes.

The discrete 0.1 mg measurement grid also generated numerous tied observations and allowed different specimen‐level distributions to produce identical group totals and arithmetic means. Consequently, the identical arithmetic means calculated for several corresponding Group A and Group B protocols should not be interpreted as evidence of identical specimen‐level responses or equivalence between the two irrigant regimens.

The descriptive optical microscopy findings showed a broadly similar pattern of material removal and therefore provide complementary, although non‐inferential, evidence. Nevertheless, microscopy does not eliminate the potential gravimetric offset, and agreement between the two methods cannot establish that the full W2–W3 difference resulted from hydrogel removal.

Within the constraints of the present model, none of the tested protocols achieved complete hydrogel removal from the smaller lateral canal. Incomplete removal has also been reported in related artificial‐canal investigations. Moreover, Retsas et al. (2022) found that ultrasonic activation improved removal of a dual‐species microbial biofilm from artificial lateral canals but did not achieve complete eradication. Because their biological model differed fundamentally from the acellular hydrogel used in the present study, this comparison should be interpreted qualitatively rather than as evidence of equivalent cleaning or antimicrobial efficacy.

One possible explanation for the incomplete removal is the conservative positioning of the activation tips. In the present study, gutta‐percha cones and sonic and ultrasonic tips were positioned 2 mm short of the working length, leaving an approximate distance of 1 mm between the tip and the entrance of the smaller lateral canal. This position was selected with reference to clinically used safety principles intended to reduce the risk of apical irrigant extrusion. Bürklein et al. (2024) demonstrated in a three‐dimensionally printed side‐canal model that tip design, energy settings and insertion depth influenced cleaning performance, while a smaller laser tip was associated with more frequent irrigant extrusion. The position selected in the present investigation may therefore have limited hydrodynamic energy transfer towards the apical lateral canal while reducing the potential risk associated with deeper tip placement.

The geometry of the lateral canals may also have contributed to the incomplete removal. Both lateral canals had considerable lengths relative to their diameters, resulting in a substantial hydrogel volume and potentially restricting irrigant exchange and shear‐stress generation. Robinson et al. (2018) demonstrated that the effectiveness of streaming and cavitation within lateral morphological features depends on local hydrodynamic conditions, while Donnermeyer et al. (2023, 2024) reported activation‐dependent differences in hydrogel removal from lateral canals and isthmuses in complex three‐dimensionally printed systems. These findings support the interpretation that both activation strategy and model geometry influenced the present results.

The greater hydrogel removal observed with the internally heated protocols is biologically plausible because heating is known to increase the tissue‐dissolving activity of sodium hypochlorite. Previous experimental studies using pulp tissue have reported faster dissolution at higher irrigant temperatures and enhanced dissolution following intracanal heating (Abdellatif, Plotino et al. 2025; Iandolo et al. 2021, 2025; Abdellatif, Cernera et al. 2025). In particular, Iandolo et al. (2021) reported significantly greater pulp‐tissue dissolution when internal heating was followed by sonic or ultrasonic activation in an isthmus model. Abdellatif, Plotino et al. (2025) similarly found markedly faster dissolution of bovine pulp tissue with heated sodium hypochlorite and heated sodium hypochlorite supplemented with Dual Rinse HEDP, whereas heated saline did not dissolve the pulp specimens.

Nevertheless, the gelatin–hyaluronan hydrogel may be more susceptible than pulp tissue to direct physical softening or liquefaction. Because no saline‐only internal‐heating group was included and temperatures within the artificial lateral canals were not measured, the present findings cannot distinguish enhanced chemical dissolution by the heated irrigant from thermally induced changes in the physical state of the hydrogel. Mechanical displacement resulting from sonic, ultrasonic, or manual dynamic activation may also have contributed. Consequently, the results represent the overall performance of the complete irrigation protocols rather than the independent effects of irrigant temperature, chemistry, or activation.

The incomplete hydrogel removal observed in the smaller apical lateral canal is consistent with the recognized difficulty of delivering irrigants and activation energy to complex apical anatomy (Boutsioukis and Arias‐Moliz 2022). Nevertheless, because the present investigation used a rigid artificial canal system and a thermosensitive acellular hydrogel, it cannot establish the clinical disinfection efficacy of the tested protocols. Further investigations using extracted teeth, biological tissue, and microbial biofilm models are required to determine whether the observed differences are reproduced under more biologically representative conditions.

## 5 Limitations

Several limitations of this study must be acknowledged. First, the use of 3D‐printed resin models, although enabling excellent standardization and reproducibility, cannot fully replicate the structural, chemical, and biological characteristics of natural dentine, including its porosity, buffering capacity, and organic composition.

Second, the gelatin–hyaluronan hydrogel provided a controlled and reproducible surrogate for organic debris but did not contain microorganisms or reproduce the extracellular polymeric matrix, spatial organization, biological activity, or resistance of an endodontic biofilm. Accordingly, the present findings relate exclusively to hydrogel removal and cannot be interpreted as evidence of antimicrobial or antibiofilm efficacy. In addition, the microscopy assessment was based on the two‐dimensional projected area of stained hydrogel within the lateral‐canal images and did not quantify the three‐dimensional volume or spatial distribution of the residual material. Consequently, a reduction in projected area cannot be interpreted as an equivalent reduction in hydrogel volume or as evidence of clinical disinfection.

Third, although enhanced organic‐tissue dissolution by heated sodium hypochlorite is well documented in studies using actual pulp tissue, the experimental hydrogel may additionally undergo direct temperature‐dependent softening or liquefaction. In the absence of a heated saline control and direct temperature measurements within the lateral canals, the extent of this model‐specific thermal contribution cannot be quantified. Accordingly, comparisons between heated and non‐heated groups should be interpreted as comparisons between complete irrigation protocols and not as evidence of the independent effect of internal heating.

Fourth, alternative activation‐tip insertion depths might have produced different hydrogel‐removal outcomes and altered the risk of apical irrigant extrusion. The present findings should therefore be interpreted strictly within the constraints of this standardized artificial model. Although the selected tip positions were based on clinically used safety principles, an acellular hydrogel within a rigid resin canal cannot reproduce the biological, chemical, and structural complexity of the clinical root canal environment.

Finally, M.C., who performed the irrigation procedures, could not be blinded to experimental‐group allocation during intervention delivery because the irrigants, activation devices, and procedures were visibly distinguishable. However, the gravimetric measurements were performed by A.A., who was not involved in randomization or intervention delivery and was blinded to group allocation. The microscopy images were independently assessed by A.I., who was not involved in randomization or the irrigation procedures and was blinded to the coding key during image analysis. Nevertheless, a formal intra‐examiner reliability coefficient for the image‐thresholding procedure could not be reconstructed from the archived examiner‐level data, and the specimen‐level microscopy measurements were not available for independent re‐analysis. The microscopy findings were therefore treated exclusively as a supportive descriptive outcome. Residual subjectivity in image segmentation and the possibility of measurement‐related detection bias cannot be completely excluded.

## 6 Conclusions

Within the limitations of this standardized thermosensitive hydrogel model, protocols combining internal heating with manual dynamic, ultrasonic or sonic activation produced greater net specimen mass loss and greater descriptive microscopy‐based hydrogel removal than conventional irrigation or single‐mode activation. However, the experimental design could not distinguish enhanced sodium‐hypochlorite‐mediated chemical dissolution from thermally induced hydrogel softening or mechanical displacement. None of the protocols completely removed the hydrogel from the smaller apical lateral canal, and the findings should not be extrapolated directly to clinical root canal systems.

## Author Contributions

All authors contributed to the study conception and design. Material preparation and data collection were performed by Alfonso Acerra, Dina Abdellatif, Mariangela Cernera, and Alfredo Iandolo. Data analysis was performed by Alfonso Acerra, Dina Abdellatif, Gianrico Spagnuolo, Davide Mancino, and Alfredo Iandolo. The first draft of the manuscript was written by Alfonso Acerra, Dina Abdellatif, Francesco Giordano, and Alfredo Iandolo. All authors commented on previous versions of the manuscript. All authors read and approved the final manuscript.

## Funding

The authors have nothing to report.

## Ethics Statement

This study was conducted in vitro and did not involve human participants or animal‐derived tissues. Therefore, ethical approval from an institutional review board or ethics committee was not required.

## Conflicts of Interest

The authors declare no conflicts of interest.

## Supporting information


Supporting File

## Data Availability

The data that support the findings of this study are available from the corresponding author upon reasonable request.
