# Supplementary material for: Comparative Evaluation of Advanced Irrigant Activation Protocols on Hydrogel Removal From Standardized Artificial Lateral Canals: An In Vitro Study
Source: Clin Exp Dent Res. 2026 Aug 26;12(4):e70442. doi: 10.1002/cre2.70442 (PMC13519040; doi:10.1002/cre2.70442)
Supplement: Supplementary file 1 [file CRE2-12-e70442-s001.docx]

**Supplementary Table S1. Pairwise comparisons of net specimen mass loss among the 16 experimental groups.**

Pairwise comparisons were performed using the Mann–Whitney U test. Z values were tie-corrected and calculated without continuity correction. Signed effect sizes were calculated as r = Z/√N; negative values indicate lower net specimen mass loss in the first-listed group, whereas positive values indicate higher values. Unadjusted percentile bootstrap 95% confidence intervals were obtained by resampling independently within each group (2,000 resamples; fixed seed 20260731). P-values were adjusted for all 120 pairwise comparisons using the Bonferroni procedure. Confidence intervals were not adjusted for multiplicity. Absolute r values of approximately 0.10, 0.30 and 0.50 were interpreted as small, moderate and large effects, respectively; values below 0.10 were classified as negligible. All comparisons are reported. Group key: A, 3% NaOCl; B, 3% NaOCl supplemented with Dual Rinse HEDP; 1, saline control; 2, conventional syringe irrigation; 3, sonic activation; 4, passive ultrasonic irrigation; 5, internal heating; 6, internal heating plus manual dynamic activation; 7, internal heating plus passive ultrasonic irrigation; 8, internal heating plus sonic activation.

| **Comparison** | **U** | **Z** | **Unadjusted p** | **Bonferroni-adjusted p** | **r** | **95% CI lower** | **95% CI upper** | **Magnitude** |
| --- | --- | --- | --- | --- | --- | --- | --- | --- |
| A1 vs A2 | 60.5 | 0.000 | 1.0000 | 1.0000 | 0.000 | 0.000 | 0.000 | Negligible |
| A1 vs A3 | 0.0 | -4.491 | <0.0001 | 0.0009 | -0.957 | -0.977 | -0.932 | Large |
| A1 vs A4 | 0.0 | -4.322 | <0.0001 | 0.0019 | -0.922 | -0.943 | -0.922 | Large |
| A1 vs A5 | 0.0 | -4.330 | <0.0001 | 0.0018 | -0.923 | -0.957 | -0.913 | Large |
| A1 vs A6 | 0.0 | -4.330 | <0.0001 | 0.0018 | -0.923 | -0.957 | -0.913 | Large |
| A1 vs A7 | 0.0 | -4.270 | <0.0001 | 0.0023 | -0.910 | -0.925 | -0.909 | Large |
| A1 vs A8 | 0.0 | -4.278 | <0.0001 | 0.0023 | -0.912 | -0.931 | -0.912 | Large |
| A1 vs B1 | 60.5 | 0.000 | 1.0000 | 1.0000 | 0.000 | 0.000 | 0.000 | Negligible |
| A1 vs B2 | 60.5 | 0.000 | 1.0000 | 1.0000 | 0.000 | 0.000 | 0.000 | Negligible |
| A1 vs B3 | 0.0 | -4.278 | <0.0001 | 0.0023 | -0.912 | -0.931 | -0.912 | Large |
| A1 vs B4 | 0.0 | -4.308 | <0.0001 | 0.0020 | -0.918 | -0.943 | -0.913 | Large |
| A1 vs B5 | 0.0 | -4.288 | <0.0001 | 0.0022 | -0.914 | -0.932 | -0.912 | Large |
| A1 vs B6 | 0.0 | -4.297 | <0.0001 | 0.0021 | -0.916 | -0.943 | -0.910 | Large |
| A1 vs B7 | 0.0 | -4.278 | <0.0001 | 0.0023 | -0.912 | -0.931 | -0.908 | Large |
| A1 vs B8 | 0.0 | -4.274 | <0.0001 | 0.0023 | -0.911 | -0.931 | -0.909 | Large |
| A2 vs A3 | 0.0 | -4.491 | <0.0001 | 0.0009 | -0.957 | -0.977 | -0.932 | Large |
| A2 vs A4 | 0.0 | -4.322 | <0.0001 | 0.0019 | -0.922 | -0.943 | -0.922 | Large |
| A2 vs A5 | 0.0 | -4.330 | <0.0001 | 0.0018 | -0.923 | -0.957 | -0.913 | Large |
| A2 vs A6 | 0.0 | -4.330 | <0.0001 | 0.0018 | -0.923 | -0.957 | -0.913 | Large |
| A2 vs A7 | 0.0 | -4.270 | <0.0001 | 0.0023 | -0.910 | -0.923 | -0.909 | Large |
| A2 vs A8 | 0.0 | -4.278 | <0.0001 | 0.0023 | -0.912 | -0.931 | -0.912 | Large |
| A2 vs B1 | 60.5 | 0.000 | 1.0000 | 1.0000 | 0.000 | 0.000 | 0.000 | Negligible |
| A2 vs B2 | 60.5 | 0.000 | 1.0000 | 1.0000 | 0.000 | 0.000 | 0.000 | Negligible |
| A2 vs B3 | 0.0 | -4.278 | <0.0001 | 0.0023 | -0.912 | -0.931 | -0.912 | Large |
| A2 vs B4 | 0.0 | -4.308 | <0.0001 | 0.0020 | -0.918 | -0.943 | -0.913 | Large |
| A2 vs B5 | 0.0 | -4.288 | <0.0001 | 0.0022 | -0.914 | -0.932 | -0.912 | Large |

**Supplementary Table S1. Pairwise comparisons of net specimen mass loss among the 16 experimental groups (continued).**

| **Comparison** | **U** | **Z** | **Unadjusted p** | **Bonferroni-adjusted p** | **r** | **95% CI lower** | **95% CI upper** | **Magnitude** |
| --- | --- | --- | --- | --- | --- | --- | --- | --- |
| A2 vs B6 | 0.0 | -4.297 | <0.0001 | 0.0021 | -0.916 | -0.943 | -0.910 | Large |
| A2 vs B7 | 0.0 | -4.278 | <0.0001 | 0.0023 | -0.912 | -0.931 | -0.909 | Large |
| A2 vs B8 | 0.0 | -4.274 | <0.0001 | 0.0023 | -0.911 | -0.931 | -0.910 | Large |
| A3 vs A4 | 38.5 | -1.871 | 0.0614 | 1.0000 | -0.399 | -0.714 | 0.000 | Moderate |
| A3 vs A5 | 21.5 | -2.963 | 0.0030 | 0.3656 | -0.632 | -0.879 | -0.357 | Large |
| A3 vs A6 | 0.0 | -4.252 | <0.0001 | 0.0025 | -0.907 | -0.943 | -0.884 | Large |
| A3 vs A7 | 0.0 | -4.195 | <0.0001 | 0.0033 | -0.894 | -0.922 | -0.874 | Large |
| A3 vs A8 | 0.0 | -4.203 | <0.0001 | 0.0032 | -0.896 | -0.923 | -0.876 | Large |
| A3 vs B1 | 121.0 | 4.491 | <0.0001 | 0.0009 | 0.957 | 0.932 | 0.977 | Large |
| A3 vs B2 | 121.0 | 4.491 | <0.0001 | 0.0009 | 0.957 | 0.932 | 0.977 | Large |
| A3 vs B3 | 69.5 | 0.691 | 0.4897 | 1.0000 | 0.147 | -0.362 | 0.577 | Small |
| A3 vs B4 | 38.0 | -1.752 | 0.0798 | 1.0000 | -0.373 | -0.739 | 0.068 | Moderate |
| A3 vs B5 | 26.5 | -2.626 | 0.0086 | 1.0000 | -0.560 | -0.802 | -0.216 | Large |
| A3 vs B6 | 0.0 | -4.221 | <0.0001 | 0.0029 | -0.900 | -0.939 | -0.877 | Large |
| A3 vs B7 | 0.0 | -4.203 | <0.0001 | 0.0032 | -0.896 | -0.931 | -0.874 | Large |
| A3 vs B8 | 1.0 | -4.134 | <0.0001 | 0.0043 | -0.881 | -0.923 | -0.818 | Large |
| A4 vs A5 | 41.5 | -1.421 | 0.1553 | 1.0000 | -0.303 | -0.651 | 0.090 | Moderate |
| A4 vs A6 | 0.0 | -4.108 | <0.0001 | 0.0048 | -0.876 | -0.911 | -0.868 | Large |
| A4 vs A7 | 0.0 | -4.057 | <0.0001 | 0.0060 | -0.865 | -0.893 | -0.864 | Large |
| A4 vs A8 | 0.0 | -4.064 | <0.0001 | 0.0058 | -0.867 | -0.894 | -0.867 | Large |
| A4 vs B1 | 121.0 | 4.322 | <0.0001 | 0.0019 | 0.922 | 0.922 | 0.943 | Large |
| A4 vs B2 | 121.0 | 4.322 | <0.0001 | 0.0019 | 0.922 | 0.922 | 0.943 | Large |
| A4 vs B3 | 83.5 | 1.635 | 0.1021 | 1.0000 | 0.348 | -0.047 | 0.695 | Moderate |
| A4 vs B4 | 58.0 | -0.186 | 0.8528 | 1.0000 | -0.040 | -0.454 | 0.380 | Negligible |
| A4 vs B5 | 44.5 | -1.165 | 0.2440 | 1.0000 | -0.248 | -0.589 | 0.131 | Small |
| A4 vs B6 | 0.0 | -4.080 | <0.0001 | 0.0054 | -0.870 | -0.905 | -0.865 | Large |
| A4 vs B7 | 0.0 | -4.064 | <0.0001 | 0.0058 | -0.867 | -0.894 | -0.864 | Large |
| A4 vs B8 | 5.0 | -3.764 | 0.0002 | 0.0200 | -0.803 | -0.886 | -0.700 | Large |
| A5 vs A6 | 0.5 | -4.082 | <0.0001 | 0.0054 | -0.870 | -0.915 | -0.832 | Large |
| A5 vs A7 | 0.0 | -4.063 | <0.0001 | 0.0058 | -0.866 | -0.896 | -0.859 | Large |
| A5 vs A8 | 2.0 | -3.948 | <0.0001 | 0.0095 | -0.842 | -0.901 | -0.771 | Large |
| A5 vs B1 | 121.0 | 4.330 | <0.0001 | 0.0018 | 0.923 | 0.913 | 0.957 | Large |

**Supplementary Table S1. Pairwise comparisons of net specimen mass loss among the 16 experimental groups (continued).**

| **Comparison** | **U** | **Z** | **Unadjusted p** | **Bonferroni-adjusted p** | **r** | **95% CI lower** | **95% CI upper** | **Magnitude** |
| --- | --- | --- | --- | --- | --- | --- | --- | --- |
| A5 vs B2 | 121.0 | 4.330 | <0.0001 | 0.0018 | 0.923 | 0.914 | 0.957 | Large |
| A5 vs B3 | 95.5 | 2.465 | 0.0137 | 1.0000 | 0.525 | 0.179 | 0.792 | Large |
| A5 vs B4 | 76.0 | 1.165 | 0.2439 | 1.0000 | 0.248 | -0.178 | 0.583 | Small |
| A5 vs B5 | 61.5 | 0.073 | 0.9416 | 1.0000 | 0.016 | -0.398 | 0.459 | Negligible |
| A5 vs B6 | 1.0 | -4.022 | <0.0001 | 0.0069 | -0.858 | -0.907 | -0.809 | Large |
| A5 vs B7 | 0.5 | -4.038 | <0.0001 | 0.0065 | -0.861 | -0.901 | -0.827 | Large |
| A5 vs B8 | 9.5 | -3.495 | 0.0005 | 0.0569 | -0.745 | -0.881 | -0.545 | Large |
| A6 vs A7 | 25.5 | -2.460 | 0.0139 | 1.0000 | -0.524 | -0.789 | -0.179 | Large |
| A6 vs A8 | 72.5 | 0.857 | 0.3913 | 1.0000 | 0.183 | -0.268 | 0.573 | Small |
| A6 vs B1 | 121.0 | 4.330 | <0.0001 | 0.0018 | 0.923 | 0.913 | 0.957 | Large |
| A6 vs B2 | 121.0 | 4.330 | <0.0001 | 0.0018 | 0.923 | 0.913 | 0.957 | Large |
| A6 vs B3 | 121.0 | 4.070 | <0.0001 | 0.0056 | 0.868 | 0.861 | 0.901 | Large |
| A6 vs B4 | 121.0 | 4.096 | <0.0001 | 0.0050 | 0.873 | 0.864 | 0.912 | Large |
| A6 vs B5 | 120.0 | 4.015 | <0.0001 | 0.0071 | 0.856 | 0.805 | 0.902 | Large |
| A6 vs B6 | 63.0 | 0.186 | 0.8526 | 1.0000 | 0.040 | -0.388 | 0.454 | Negligible |
| A6 vs B7 | 24.5 | -2.489 | 0.0128 | 1.0000 | -0.531 | -0.823 | -0.150 | Large |
| A6 vs B8 | 64.5 | 0.286 | 0.7748 | 1.0000 | 0.061 | -0.387 | 0.481 | Negligible |
| A7 vs A8 | 100.0 | 2.691 | 0.0071 | 0.8542 | 0.574 | 0.291 | 0.799 | Large |
| A7 vs B1 | 121.0 | 4.270 | <0.0001 | 0.0023 | 0.910 | 0.909 | 0.925 | Large |
| A7 vs B2 | 121.0 | 4.270 | <0.0001 | 0.0023 | 0.910 | 0.909 | 0.923 | Large |
| A7 vs B3 | 121.0 | 4.021 | <0.0001 | 0.0070 | 0.857 | 0.857 | 0.876 | Large |
| A7 vs B4 | 121.0 | 4.045 | <0.0001 | 0.0063 | 0.862 | 0.859 | 0.888 | Large |
| A7 vs B5 | 121.0 | 4.029 | <0.0001 | 0.0067 | 0.859 | 0.858 | 0.882 | Large |
| A7 vs B6 | 93.5 | 2.276 | 0.0228 | 1.0000 | 0.485 | 0.123 | 0.760 | Moderate |
| A7 vs B7 | 58.5 | -0.138 | 0.8903 | 1.0000 | -0.029 | -0.447 | 0.380 | Negligible |
| A7 vs B8 | 95.0 | 2.364 | 0.0181 | 1.0000 | 0.504 | 0.164 | 0.757 | Large |
| A8 vs B1 | 121.0 | 4.278 | <0.0001 | 0.0023 | 0.912 | 0.912 | 0.931 | Large |
| A8 vs B2 | 121.0 | 4.278 | <0.0001 | 0.0023 | 0.912 | 0.912 | 0.931 | Large |
| A8 vs B3 | 121.0 | 4.028 | <0.0001 | 0.0068 | 0.859 | 0.859 | 0.880 | Large |
| A8 vs B4 | 121.0 | 4.052 | <0.0001 | 0.0061 | 0.864 | 0.861 | 0.892 | Large |
| A8 vs B5 | 117.0 | 3.796 | 0.0001 | 0.0177 | 0.809 | 0.722 | 0.879 | Large |
| A8 vs B6 | 51.0 | -0.667 | 0.5050 | 1.0000 | -0.142 | -0.528 | 0.279 | Small |

**Supplementary Table S1. Pairwise comparisons of net specimen mass loss among the 16 experimental groups (continued).**

| **Comparison** | **U** | **Z** | **Unadjusted p** | **Bonferroni-adjusted p** | **r** | **95% CI lower** | **95% CI upper** | **Magnitude** |
| --- | --- | --- | --- | --- | --- | --- | --- | --- |
| A8 vs B7 | 21.5 | -2.659 | 0.0078 | 0.9410 | -0.567 | -0.816 | -0.260 | Large |
| A8 vs B8 | 57.0 | -0.241 | 0.8095 | 1.0000 | -0.051 | -0.457 | 0.349 | Negligible |
| B1 vs B2 | 60.5 | 0.000 | 1.0000 | 1.0000 | 0.000 | 0.000 | 0.000 | Negligible |
| B1 vs B3 | 0.0 | -4.278 | <0.0001 | 0.0023 | -0.912 | -0.931 | -0.912 | Large |
| B1 vs B4 | 0.0 | -4.308 | <0.0001 | 0.0020 | -0.918 | -0.943 | -0.913 | Large |
| B1 vs B5 | 0.0 | -4.288 | <0.0001 | 0.0022 | -0.914 | -0.932 | -0.912 | Large |
| B1 vs B6 | 0.0 | -4.297 | <0.0001 | 0.0021 | -0.916 | -0.943 | -0.910 | Large |
| B1 vs B7 | 0.0 | -4.278 | <0.0001 | 0.0023 | -0.912 | -0.931 | -0.908 | Large |
| B1 vs B8 | 0.0 | -4.274 | <0.0001 | 0.0023 | -0.911 | -0.931 | -0.910 | Large |
| B2 vs B3 | 0.0 | -4.278 | <0.0001 | 0.0023 | -0.912 | -0.931 | -0.912 | Large |
| B2 vs B4 | 0.0 | -4.308 | <0.0001 | 0.0020 | -0.918 | -0.943 | -0.913 | Large |
| B2 vs B5 | 0.0 | -4.288 | <0.0001 | 0.0022 | -0.914 | -0.942 | -0.912 | Large |
| B2 vs B6 | 0.0 | -4.297 | <0.0001 | 0.0021 | -0.916 | -0.943 | -0.910 | Large |
| B2 vs B7 | 0.0 | -4.278 | <0.0001 | 0.0023 | -0.912 | -0.931 | -0.908 | Large |
| B2 vs B8 | 0.0 | -4.274 | <0.0001 | 0.0023 | -0.911 | -0.931 | -0.910 | Large |
| B3 vs B4 | 38.0 | -1.581 | 0.1139 | 1.0000 | -0.337 | -0.709 | 0.061 | Moderate |
| B3 vs B5 | 27.5 | -2.286 | 0.0223 | 1.0000 | -0.487 | -0.769 | -0.143 | Moderate |
| B3 vs B6 | 0.0 | -4.043 | <0.0001 | 0.0063 | -0.862 | -0.889 | -0.858 | Large |
| B3 vs B7 | 0.0 | -4.028 | <0.0001 | 0.0068 | -0.859 | -0.883 | -0.856 | Large |
| B3 vs B8 | 3.0 | -3.841 | 0.0001 | 0.0147 | -0.819 | -0.875 | -0.741 | Large |
| B4 vs B5 | 47.0 | -0.974 | 0.3299 | 1.0000 | -0.208 | -0.581 | 0.205 | Small |
| B4 vs B6 | 0.0 | -4.068 | <0.0001 | 0.0057 | -0.867 | -0.901 | -0.860 | Large |
| B4 vs B7 | 0.0 | -4.052 | <0.0001 | 0.0061 | -0.864 | -0.894 | -0.859 | Large |
| B4 vs B8 | 6.0 | -3.700 | 0.0002 | 0.0259 | -0.789 | -0.880 | -0.683 | Large |
| B5 vs B6 | 2.0 | -3.927 | <0.0001 | 0.0103 | -0.837 | -0.890 | -0.781 | Large |
| B5 vs B7 | 1.0 | -3.973 | <0.0001 | 0.0085 | -0.847 | -0.885 | -0.801 | Large |
| B5 vs B8 | 10.0 | -3.404 | 0.0007 | 0.0798 | -0.726 | -0.871 | -0.518 | Large |
| B6 vs B7 | 27.5 | -2.248 | 0.0246 | 1.0000 | -0.479 | -0.800 | -0.119 | Moderate |
| B6 vs B8 | 64.0 | 0.244 | 0.8069 | 1.0000 | 0.052 | -0.372 | 0.463 | Negligible |
| B7 vs B8 | 96.5 | 2.468 | 0.0136 | 1.0000 | 0.526 | 0.196 | 0.782 | Large |

**Supplementary Table S2. Specimen-level gravimetric measurements and derived outcomes for the 16 experimental groups.**

W1 represents the empty-block mass, W2 the mass after hydrogel placement and solidification, and W3 the post-procedural mass. W2 − W1 represents the initial hydrogel-associated mass, whereas W2 − W3 represents net specimen mass loss. The normalised loss ratio was calculated as (W2 − W3)/(W2 − W1) and was used only in the exploratory sensitivity analysis. Source measurements and mass differences are reported to the 0.1 mg readability of the balance; the ratio is displayed to three decimal places as a derived quantity and does not imply additional measurement precision. A W2 − W3 value of 0.0 mg indicates no detectable change at 0.1 mg resolution and does not exclude smaller positive or negative fluctuations. No substitution or imputation was applied.

**Groups A1–A2**

| **Group** | **Specimen** | **W1 (mg)** | **W2 (mg)** | **W3 (mg)** | **W2 − W1 (mg)** | **W2 − W3 (mg)** | **Normalised loss ratio** |
| --- | --- | --- | --- | --- | --- | --- | --- |
| A1 | 1 | 247.0 | 247.8 | 247.8 | 0.8 | 0.0 | 0.000 |
| A1 | 2 | 247.0 | 247.8 | 247.8 | 0.8 | 0.0 | 0.000 |
| A1 | 3 | 247.0 | 247.8 | 247.8 | 0.8 | 0.0 | 0.000 |
| A1 | 4 | 247.0 | 247.8 | 247.8 | 0.8 | 0.0 | 0.000 |
| A1 | 5 | 247.0 | 247.8 | 247.8 | 0.8 | 0.0 | 0.000 |
| A1 | 6 | 247.0 | 247.8 | 247.8 | 0.8 | 0.0 | 0.000 |
| A1 | 7 | 247.0 | 247.8 | 247.8 | 0.8 | 0.0 | 0.000 |
| A1 | 8 | 247.0 | 247.8 | 247.8 | 0.8 | 0.0 | 0.000 |
| A1 | 9 | 247.0 | 247.8 | 247.8 | 0.8 | 0.0 | 0.000 |
| A1 | 10 | 247.0 | 247.8 | 247.8 | 0.8 | 0.0 | 0.000 |
| A1 | 11 | 247.0 | 247.8 | 247.8 | 0.8 | 0.0 | 0.000 |
| A2 | 1 | 247.0 | 247.8 | 247.8 | 0.8 | 0.0 | 0.000 |
| A2 | 2 | 247.0 | 247.8 | 247.8 | 0.8 | 0.0 | 0.000 |
| A2 | 3 | 247.0 | 247.8 | 247.8 | 0.8 | 0.0 | 0.000 |
| A2 | 4 | 247.0 | 247.8 | 247.8 | 0.8 | 0.0 | 0.000 |
| A2 | 5 | 247.0 | 247.8 | 247.8 | 0.8 | 0.0 | 0.000 |
| A2 | 6 | 247.0 | 247.8 | 247.8 | 0.8 | 0.0 | 0.000 |
| A2 | 7 | 247.0 | 247.8 | 247.8 | 0.8 | 0.0 | 0.000 |
| A2 | 8 | 247.0 | 247.8 | 247.8 | 0.8 | 0.0 | 0.000 |
| A2 | 9 | 247.0 | 247.8 | 247.8 | 0.8 | 0.0 | 0.000 |
| A2 | 10 | 247.0 | 247.8 | 247.8 | 0.8 | 0.0 | 0.000 |
| A2 | 11 | 247.0 | 247.8 | 247.8 | 0.8 | 0.0 | 0.000 |

**Supplementary Table S2. Specimen-level gravimetric measurements and derived outcomes for the 16 experimental groups (continued).**

**Groups A3–A4**

| **Group** | **Specimen** | **W1 (mg)** | **W2 (mg)** | **W3 (mg)** | **W2 − W1 (mg)** | **W2 − W3 (mg)** | **Normalised loss ratio** |
| --- | --- | --- | --- | --- | --- | --- | --- |
| A3 | 1 | 247.0 | 247.8 | 247.6 | 0.8 | 0.2 | 0.250 |
| A3 | 2 | 247.0 | 247.9 | 247.7 | 0.9 | 0.2 | 0.222 |
| A3 | 3 | 247.0 | 247.8 | 247.6 | 0.8 | 0.2 | 0.250 |
| A3 | 4 | 247.0 | 247.8 | 247.6 | 0.8 | 0.2 | 0.250 |
| A3 | 5 | 247.0 | 247.8 | 247.6 | 0.8 | 0.2 | 0.250 |
| A3 | 6 | 247.0 | 247.8 | 247.6 | 0.8 | 0.2 | 0.250 |
| A3 | 7 | 247.0 | 247.8 | 247.6 | 0.8 | 0.2 | 0.250 |
| A3 | 8 | 247.0 | 247.9 | 247.6 | 0.9 | 0.3 | 0.333 |
| A3 | 9 | 247.0 | 247.8 | 247.6 | 0.8 | 0.2 | 0.250 |
| A3 | 10 | 247.0 | 247.8 | 247.6 | 0.8 | 0.2 | 0.250 |
| A3 | 11 | 247.0 | 247.9 | 247.7 | 0.9 | 0.2 | 0.222 |
| A4 | 1 | 247.0 | 247.8 | 247.6 | 0.8 | 0.2 | 0.250 |
| A4 | 2 | 247.0 | 247.8 | 247.5 | 0.8 | 0.3 | 0.375 |
| A4 | 3 | 247.0 | 247.9 | 247.6 | 0.9 | 0.3 | 0.333 |
| A4 | 4 | 247.0 | 247.8 | 247.6 | 0.8 | 0.2 | 0.250 |
| A4 | 5 | 247.0 | 247.8 | 247.5 | 0.8 | 0.3 | 0.375 |
| A4 | 6 | 247.0 | 247.9 | 247.6 | 0.9 | 0.3 | 0.333 |
| A4 | 7 | 247.0 | 247.8 | 247.5 | 0.8 | 0.3 | 0.375 |
| A4 | 8 | 247.0 | 247.8 | 247.6 | 0.8 | 0.2 | 0.250 |
| A4 | 9 | 247.0 | 247.8 | 247.6 | 0.8 | 0.2 | 0.250 |
| A4 | 10 | 247.0 | 247.8 | 247.6 | 0.8 | 0.2 | 0.250 |
| A4 | 11 | 247.0 | 247.8 | 247.6 | 0.8 | 0.2 | 0.250 |

**Supplementary Table S2. Specimen-level gravimetric measurements and derived outcomes for the 16 experimental groups (continued).**

**Groups A5–A6**

| **Group** | **Specimen** | **W1 (mg)** | **W2 (mg)** | **W3 (mg)** | **W2 − W1 (mg)** | **W2 − W3 (mg)** | **Normalised loss ratio** |
| --- | --- | --- | --- | --- | --- | --- | --- |
| A5 | 1 | 247.0 | 247.8 | 247.6 | 0.8 | 0.2 | 0.250 |
| A5 | 2 | 247.0 | 247.8 | 247.6 | 0.8 | 0.2 | 0.250 |
| A5 | 3 | 247.0 | 247.9 | 247.6 | 0.9 | 0.3 | 0.333 |
| A5 | 4 | 247.0 | 247.8 | 247.5 | 0.8 | 0.3 | 0.375 |
| A5 | 5 | 247.0 | 247.8 | 247.5 | 0.8 | 0.3 | 0.375 |
| A5 | 6 | 247.0 | 247.8 | 247.6 | 0.8 | 0.2 | 0.250 |
| A5 | 7 | 247.0 | 247.8 | 247.5 | 0.8 | 0.3 | 0.375 |
| A5 | 8 | 247.0 | 247.8 | 247.5 | 0.8 | 0.3 | 0.375 |
| A5 | 9 | 247.0 | 247.8 | 247.5 | 0.8 | 0.3 | 0.375 |
| A5 | 10 | 247.0 | 247.8 | 247.4 | 0.8 | 0.4 | 0.500 |
| A5 | 11 | 247.0 | 247.8 | 247.5 | 0.8 | 0.3 | 0.375 |
| A6 | 1 | 247.0 | 247.8 | 247.3 | 0.8 | 0.5 | 0.625 |
| A6 | 2 | 247.0 | 247.8 | 247.3 | 0.8 | 0.5 | 0.625 |
| A6 | 3 | 247.0 | 247.8 | 247.2 | 0.8 | 0.6 | 0.750 |
| A6 | 4 | 247.0 | 247.8 | 247.3 | 0.8 | 0.5 | 0.625 |
| A6 | 5 | 247.0 | 247.8 | 247.3 | 0.8 | 0.5 | 0.625 |
| A6 | 6 | 247.0 | 247.8 | 247.3 | 0.8 | 0.5 | 0.625 |
| A6 | 7 | 247.0 | 247.8 | 247.2 | 0.8 | 0.6 | 0.750 |
| A6 | 8 | 247.0 | 247.8 | 247.2 | 0.8 | 0.6 | 0.750 |
| A6 | 9 | 247.0 | 247.8 | 247.3 | 0.8 | 0.5 | 0.625 |
| A6 | 10 | 247.0 | 247.8 | 247.4 | 0.8 | 0.4 | 0.500 |
| A6 | 11 | 247.0 | 247.8 | 247.3 | 0.8 | 0.5 | 0.625 |

**Supplementary Table S2. Specimen-level gravimetric measurements and derived outcomes for the 16 experimental groups (continued).**

**Groups A7–A8**

| **Group** | **Specimen** | **W1 (mg)** | **W2 (mg)** | **W3 (mg)** | **W2 − W1 (mg)** | **W2 − W3 (mg)** | **Normalised loss ratio** |
| --- | --- | --- | --- | --- | --- | --- | --- |
| A7 | 1 | 247.0 | 247.8 | 247.2 | 0.8 | 0.6 | 0.750 |
| A7 | 2 | 247.0 | 247.8 | 247.3 | 0.8 | 0.5 | 0.625 |
| A7 | 3 | 247.0 | 247.8 | 247.1 | 0.8 | 0.7 | 0.875 |
| A7 | 4 | 247.0 | 247.8 | 247.2 | 0.8 | 0.6 | 0.750 |
| A7 | 5 | 247.0 | 247.8 | 247.3 | 0.8 | 0.5 | 0.625 |
| A7 | 6 | 247.0 | 247.8 | 247.3 | 0.8 | 0.5 | 0.625 |
| A7 | 7 | 247.0 | 247.8 | 247.2 | 0.8 | 0.6 | 0.750 |
| A7 | 8 | 247.0 | 247.8 | 247.1 | 0.8 | 0.7 | 0.875 |
| A7 | 9 | 247.0 | 247.8 | 247.0 | 0.8 | 0.8 | 1.000 |
| A7 | 10 | 247.0 | 247.8 | 247.2 | 0.8 | 0.6 | 0.750 |
| A7 | 11 | 247.0 | 247.8 | 247.1 | 0.8 | 0.7 | 0.875 |
| A8 | 1 | 247.0 | 247.8 | 247.4 | 0.8 | 0.4 | 0.500 |
| A8 | 2 | 247.0 | 247.8 | 247.4 | 0.8 | 0.4 | 0.500 |
| A8 | 3 | 247.0 | 247.8 | 247.2 | 0.8 | 0.6 | 0.750 |
| A8 | 4 | 247.0 | 247.8 | 247.3 | 0.8 | 0.5 | 0.625 |
| A8 | 5 | 247.0 | 247.8 | 247.3 | 0.8 | 0.5 | 0.625 |
| A8 | 6 | 247.0 | 247.8 | 247.3 | 0.8 | 0.5 | 0.625 |
| A8 | 7 | 247.0 | 247.8 | 247.2 | 0.8 | 0.6 | 0.750 |
| A8 | 8 | 247.0 | 247.8 | 247.2 | 0.8 | 0.6 | 0.750 |
| A8 | 9 | 247.0 | 247.8 | 247.3 | 0.8 | 0.5 | 0.625 |
| A8 | 10 | 247.0 | 247.8 | 247.4 | 0.8 | 0.4 | 0.500 |
| A8 | 11 | 247.0 | 247.8 | 247.4 | 0.8 | 0.4 | 0.500 |

**Supplementary Table S2. Specimen-level gravimetric measurements and derived outcomes for the 16 experimental groups (continued).**

**Groups B1–B2**

| **Group** | **Specimen** | **W1 (mg)** | **W2 (mg)** | **W3 (mg)** | **W2 − W1 (mg)** | **W2 − W3 (mg)** | **Normalised loss ratio** |
| --- | --- | --- | --- | --- | --- | --- | --- |
| B1 | 1 | 247.0 | 247.8 | 247.8 | 0.8 | 0.0 | 0.000 |
| B1 | 2 | 247.0 | 247.8 | 247.8 | 0.8 | 0.0 | 0.000 |
| B1 | 3 | 247.0 | 247.8 | 247.8 | 0.8 | 0.0 | 0.000 |
| B1 | 4 | 247.0 | 247.8 | 247.8 | 0.8 | 0.0 | 0.000 |
| B1 | 5 | 247.0 | 247.8 | 247.8 | 0.8 | 0.0 | 0.000 |
| B1 | 6 | 247.0 | 247.8 | 247.8 | 0.8 | 0.0 | 0.000 |
| B1 | 7 | 247.0 | 247.8 | 247.8 | 0.8 | 0.0 | 0.000 |
| B1 | 8 | 247.0 | 247.8 | 247.8 | 0.8 | 0.0 | 0.000 |
| B1 | 9 | 247.0 | 247.8 | 247.8 | 0.8 | 0.0 | 0.000 |
| B1 | 10 | 247.0 | 247.8 | 247.8 | 0.8 | 0.0 | 0.000 |
| B1 | 11 | 247.0 | 247.8 | 247.8 | 0.8 | 0.0 | 0.000 |
| B2 | 1 | 247.0 | 247.8 | 247.8 | 0.8 | 0.0 | 0.000 |
| B2 | 2 | 247.0 | 247.8 | 247.8 | 0.8 | 0.0 | 0.000 |
| B2 | 3 | 247.0 | 247.8 | 247.8 | 0.8 | 0.0 | 0.000 |
| B2 | 4 | 247.0 | 247.8 | 247.8 | 0.8 | 0.0 | 0.000 |
| B2 | 5 | 247.0 | 247.8 | 247.8 | 0.8 | 0.0 | 0.000 |
| B2 | 6 | 247.0 | 247.8 | 247.8 | 0.8 | 0.0 | 0.000 |
| B2 | 7 | 247.0 | 247.8 | 247.8 | 0.8 | 0.0 | 0.000 |
| B2 | 8 | 247.0 | 247.8 | 247.8 | 0.8 | 0.0 | 0.000 |
| B2 | 9 | 247.0 | 247.8 | 247.8 | 0.8 | 0.0 | 0.000 |
| B2 | 10 | 247.0 | 247.8 | 247.8 | 0.8 | 0.0 | 0.000 |
| B2 | 11 | 247.0 | 247.8 | 247.8 | 0.8 | 0.0 | 0.000 |

**Supplementary Table S2. Specimen-level gravimetric measurements and derived outcomes for the 16 experimental groups (continued).**

**Groups B3–B4**

| **Group** | **Specimen** | **W1 (mg)** | **W2 (mg)** | **W3 (mg)** | **W2 − W1 (mg)** | **W2 − W3 (mg)** | **Normalised loss ratio** |
| --- | --- | --- | --- | --- | --- | --- | --- |
| B3 | 1 | 247.0 | 247.8 | 247.5 | 0.8 | 0.3 | 0.375 |
| B3 | 2 | 247.0 | 247.9 | 247.8 | 0.9 | 0.1 | 0.111 |
| B3 | 3 | 247.0 | 247.8 | 247.5 | 0.8 | 0.3 | 0.375 |
| B3 | 4 | 247.0 | 247.8 | 247.7 | 0.8 | 0.1 | 0.125 |
| B3 | 5 | 247.0 | 247.8 | 247.6 | 0.8 | 0.2 | 0.250 |
| B3 | 6 | 247.0 | 247.8 | 247.6 | 0.8 | 0.2 | 0.250 |
| B3 | 7 | 247.0 | 247.8 | 247.6 | 0.8 | 0.2 | 0.250 |
| B3 | 8 | 247.0 | 247.9 | 247.6 | 0.9 | 0.3 | 0.333 |
| B3 | 9 | 247.0 | 247.8 | 247.6 | 0.8 | 0.2 | 0.250 |
| B3 | 10 | 247.0 | 247.8 | 247.7 | 0.8 | 0.1 | 0.125 |
| B3 | 11 | 247.0 | 247.9 | 247.8 | 0.9 | 0.1 | 0.111 |
| B4 | 1 | 247.0 | 247.8 | 247.5 | 0.8 | 0.3 | 0.375 |
| B4 | 2 | 247.0 | 247.8 | 247.6 | 0.8 | 0.2 | 0.250 |
| B4 | 3 | 247.0 | 247.9 | 247.6 | 0.9 | 0.3 | 0.333 |
| B4 | 4 | 247.0 | 247.8 | 247.6 | 0.8 | 0.2 | 0.250 |
| B4 | 5 | 247.0 | 247.8 | 247.5 | 0.8 | 0.3 | 0.375 |
| B4 | 6 | 247.0 | 247.9 | 247.6 | 0.9 | 0.3 | 0.333 |
| B4 | 7 | 247.0 | 247.8 | 247.5 | 0.8 | 0.3 | 0.375 |
| B4 | 8 | 247.0 | 247.8 | 247.6 | 0.8 | 0.2 | 0.250 |
| B4 | 9 | 247.0 | 247.8 | 247.6 | 0.8 | 0.2 | 0.250 |
| B4 | 10 | 247.0 | 247.8 | 247.5 | 0.8 | 0.3 | 0.375 |
| B4 | 11 | 247.0 | 247.8 | 247.7 | 0.8 | 0.1 | 0.125 |

**Supplementary Table S2. Specimen-level gravimetric measurements and derived outcomes for the 16 experimental groups (continued).**

**Groups B5–B6**

| **Group** | **Specimen** | **W1 (mg)** | **W2 (mg)** | **W3 (mg)** | **W2 − W1 (mg)** | **W2 − W3 (mg)** | **Normalised loss ratio** |
| --- | --- | --- | --- | --- | --- | --- | --- |
| B5 | 1 | 247.0 | 247.8 | 247.6 | 0.8 | 0.2 | 0.250 |
| B5 | 2 | 247.0 | 247.8 | 247.6 | 0.8 | 0.2 | 0.250 |
| B5 | 3 | 247.0 | 247.9 | 247.6 | 0.9 | 0.3 | 0.333 |
| B5 | 4 | 247.0 | 247.8 | 247.5 | 0.8 | 0.3 | 0.375 |
| B5 | 5 | 247.0 | 247.8 | 247.6 | 0.8 | 0.2 | 0.250 |
| B5 | 6 | 247.0 | 247.8 | 247.5 | 0.8 | 0.3 | 0.375 |
| B5 | 7 | 247.0 | 247.8 | 247.4 | 0.8 | 0.4 | 0.500 |
| B5 | 8 | 247.0 | 247.8 | 247.6 | 0.8 | 0.2 | 0.250 |
| B5 | 9 | 247.0 | 247.8 | 247.5 | 0.8 | 0.3 | 0.375 |
| B5 | 10 | 247.0 | 247.8 | 247.5 | 0.8 | 0.3 | 0.375 |
| B5 | 11 | 247.0 | 247.8 | 247.4 | 0.8 | 0.4 | 0.500 |
| B6 | 1 | 247.0 | 247.8 | 247.4 | 0.8 | 0.4 | 0.500 |
| B6 | 2 | 247.0 | 247.8 | 247.2 | 0.8 | 0.6 | 0.750 |
| B6 | 3 | 247.0 | 247.8 | 247.2 | 0.8 | 0.6 | 0.750 |
| B6 | 4 | 247.0 | 247.8 | 247.3 | 0.8 | 0.5 | 0.625 |
| B6 | 5 | 247.0 | 247.8 | 247.3 | 0.8 | 0.5 | 0.625 |
| B6 | 6 | 247.0 | 247.8 | 247.3 | 0.8 | 0.5 | 0.625 |
| B6 | 7 | 247.0 | 247.8 | 247.3 | 0.8 | 0.5 | 0.625 |
| B6 | 8 | 247.0 | 247.8 | 247.1 | 0.8 | 0.7 | 0.875 |
| B6 | 9 | 247.0 | 247.8 | 247.3 | 0.8 | 0.5 | 0.625 |
| B6 | 10 | 247.0 | 247.8 | 247.4 | 0.8 | 0.4 | 0.500 |
| B6 | 11 | 247.0 | 247.8 | 247.3 | 0.8 | 0.5 | 0.625 |

**Supplementary Table S2. Specimen-level gravimetric measurements and derived outcomes for the 16 experimental groups (continued).**

**Groups B7–B8**

| **Group** | **Specimen** | **W1 (mg)** | **W2 (mg)** | **W3 (mg)** | **W2 − W1 (mg)** | **W2 − W3 (mg)** | **Normalised loss ratio** |
| --- | --- | --- | --- | --- | --- | --- | --- |
| B7 | 1 | 247.0 | 247.8 | 247.2 | 0.8 | 0.6 | 0.750 |
| B7 | 2 | 247.0 | 247.8 | 247.3 | 0.8 | 0.5 | 0.625 |
| B7 | 3 | 247.0 | 247.8 | 247.1 | 0.8 | 0.7 | 0.875 |
| B7 | 4 | 247.0 | 247.8 | 247.2 | 0.8 | 0.6 | 0.750 |
| B7 | 5 | 247.0 | 247.8 | 247.2 | 0.8 | 0.6 | 0.750 |
| B7 | 6 | 247.0 | 247.8 | 247.4 | 0.8 | 0.4 | 0.500 |
| B7 | 7 | 247.0 | 247.8 | 247.1 | 0.8 | 0.7 | 0.875 |
| B7 | 8 | 247.0 | 247.8 | 247.2 | 0.8 | 0.6 | 0.750 |
| B7 | 9 | 247.0 | 247.8 | 247.0 | 0.8 | 0.8 | 1.000 |
| B7 | 10 | 247.0 | 247.8 | 247.2 | 0.8 | 0.6 | 0.750 |
| B7 | 11 | 247.0 | 247.8 | 247.1 | 0.8 | 0.7 | 0.875 |
| B8 | 1 | 247.0 | 247.8 | 247.3 | 0.8 | 0.5 | 0.625 |
| B8 | 2 | 247.0 | 247.8 | 247.5 | 0.8 | 0.3 | 0.375 |
| B8 | 3 | 247.0 | 247.8 | 247.2 | 0.8 | 0.6 | 0.750 |
| B8 | 4 | 247.0 | 247.8 | 247.3 | 0.8 | 0.5 | 0.625 |
| B8 | 5 | 247.0 | 247.8 | 247.3 | 0.8 | 0.5 | 0.625 |
| B8 | 6 | 247.0 | 247.8 | 247.2 | 0.8 | 0.6 | 0.750 |
| B8 | 7 | 247.0 | 247.8 | 247.3 | 0.8 | 0.5 | 0.625 |
| B8 | 8 | 247.0 | 247.8 | 247.2 | 0.8 | 0.6 | 0.750 |
| B8 | 9 | 247.0 | 247.8 | 247.2 | 0.8 | 0.6 | 0.750 |
| B8 | 10 | 247.0 | 247.8 | 247.5 | 0.8 | 0.3 | 0.375 |
| B8 | 11 | 247.0 | 247.8 | 247.4 | 0.8 | 0.4 | 0.500 |
